# Targeting phase separation on enhancers induced by transcription factor complex formations as a new strategy for treating drug-resistant cancers

**DOI:** 10.3389/fonc.2022.1024600

**Published:** 2022-10-03

**Authors:** Ken-ichi Takayama, Satoshi Inoue

**Affiliations:** ^1^ Department of Systems Aging Science and Medicine, Tokyo Metropolitan Institute of Gerontology, Tokyo, Japan; ^2^ Division of Systems Medicine and Gene Therapy, Saitama Medical University, Saitama, Japan

**Keywords:** super-enhancer, liquid-liquid phase separation, androgen receptor, prostate cancer, OCT4, nuclear analog, epigenome, collaborative transcription factor

## Abstract

The limited options for treating patients with drug-resistant cancers have emphasized the need to identify alternative treatment targets. Tumor cells have large super-enhancers (SEs) in the vicinity of important oncogenes for activation. The physical process of liquid-liquid phase separation (LLPS) contributes to the assembly of several membrane-less organelles in mammalian cells. Intrinsically disordered regions (IDRs) of proteins induce LLPS formation by developing condensates. It was discovered that key transcription factors (TFs) undergo LLPS in SEs. In addition, TFs play critical roles in the epigenetic and genetic regulation of cancer progression. Recently, we revealed the essential role of disease-specific TF collaboration changes in advanced prostate cancer (PC). OCT4 confers epigenetic changes by promoting complex formation with TFs, such as Forkhead box protein A1 (FOXA1), androgen receptor (AR) and Nuclear respiratory factor 1 (NRF1), inducing PC progression. It was demonstrated that TF collaboration through LLPS underlying transcriptional activation contributes to cancer aggressiveness and drug resistance. Moreover, the disruption of TF-mediated LLPS inhibited treatment-resistant PC tumor growth. Therefore, we propose that repression of TF collaborations involved in the LLPS of SEs could be a promising strategy for advanced cancer therapy. In this article, we summarize recent evidence highlighting the formation of LLPS on enhancers as a potent therapeutic target in advanced cancers.

## Introduction

Gene transcription is mediated by interactions among the general transcription machinery in promoter regions and gene-specific factors bound to distal regulatory enhancer regions. The formation of a functional pre-initiation complex in promoters involves the ordered stepwise assembly of RNA polymerase (pol) II and general transcription machinery that includes TFIIA, TFIIB, TFIID, TFIIE, TFIIF, TFIIH (1). Transcription factor (TF)-dependent transcription from DNA templates requires additional cofactors for epigenetic regulation and the multisubunit mediator complex. This serves as a bridge between regulatory factors at enhancers and the general transcription machinery at promoters to facilitate loop formation *via* chromatin remodeling (2). However, a model of how genes are expressed in cells and how RNA pol II produces RNA in the genome is still under development. While there is a model in which RNA pol II moves on the genome to produce RNA, there is also a model called a “transcriptional factory” in which DNA interacts or is taken up at the location where RNA pol II is locally accumulated to produce RNA (3, 4). Functional analysis of subclasses of enhancers, which are essential for activating cell-type-specific genes called super-enhancers (SEs), has shown that the aggregation or phase separation of transcriptional regulators may be important for gene regulation (5).

The physiological process of liquid-liquid phase separation (LLPS) supports the assembly of membrane-less organelles in cells. LLPS spontaneously drives the separation of a mixed solution into two or more phases of different concentrations: a dilute phase and a dense phase (6). The physical features of biomolecular condensates are essential to distinct cellular functions such as biocondensates form biochemical reactions, signaling nodes and the nuclear architecture. Intrinsically disordered regions (IDRs) of proteins induce LLPS formation by influencing the interaction with different components of the complex (7). The key property of proteins that undergo LLPS is multivalency in interacting with partners (8). IDRs produces such multivalent and weak interactions between proteins and nucleic acids. Such interactions are important to ensure the accurate regulation of biological activities. These interactions also play a pivotal role in concentrating components at discrete cellular sites.

During the development and progression of cancer, cancer cells acquire treatment-resistant and aggressive phenotypes through a variety of mechanisms (9). In the last decade, advances in next-generation sequencing and molecular biology techniques have made it possible to analyze large numbers of samples simultaneously (10). TFs have been shown to play an important role in cancer progression (10–12). For example, the emergence of distinct transcription factor complexes other than the androgen receptor (AR) has been reported in prostate cancer (PC) progression (11–14). They act as drivers of treatment resistance and have become a potential therapeutic target for PC (15). Moreover, it has become clear that TFs such as AR construct chromatin structures as large transcriptional activators (16). Phenomena due to the physical properties of proteins, such as LLPS and SE formation, have been demonstrated to be involved in the transcriptional regulation (5). It was demonstrated that the activation domains of some TFs are supposed to be IDRs and bind to the mediator complex in multiple conformations, leading to fuzzy protein-protein interactions, which is important for transcriptional activation (5). Although the importance of these new concepts in cancer has been demonstrated, more detailed mechanisms remain to be elucidated. In this paper, we review the recent evidence that has led to the increased attention on phase separation and the functional analysis of SEs. In particular, we focused on the characteristics of nuclear receptors, including the AR and its associated TFs, that promote treatment resistance in PC.

## Enhancers and super-enhancers

### Identification of super-enhnacers

**Figure 1:**
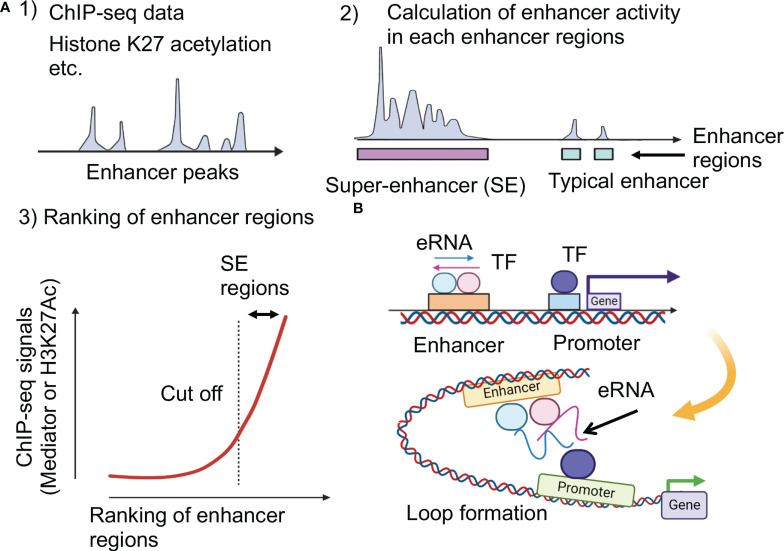
Super-enhancer and enhancer RNA (eRNA). **(A)** Identification of super-enhancers (16). The higher density of transcriptional regulators through cooperative binding to genomic regions contribute to both activated histone acetylation and higher transcriptional output at super-enhancers. K27: lysine 27. **(B)** Impacts of enhancer RNAs (eRNAs) at super-enhancers. Highly transcribed eRNAs facilitate formation of loop between promoters and enhancers.

The expression of genes specific to various cell types is regulated by enhancers distant from these genes. Using Chromatin Immunoprecipitation Sequencing (ChIP-seq), such as transcription factors and histone modifications in various types of cells, thousands of enhancer regions have been identified (2). The function of enhancers that regulate the gene expression program has been extensively studied in murine embryonic stem cells (ESCs). The co-occupancy of murine ESC genomic regions by important transcription factors Sox2, Oct4, and Nanog is correlated with enhancer activity (17, 18). Furthermore, it was clarified that some enhancers identified using ChIP-seq datasets form unusual enhancers in the vicinity of essential genes that cause diseases and regulate the pluripotency of cells (19). These enhancers are called SEs, and a unique SE is activated when cells differentiate into nerves, muscles, and other cells (20). About 200-600 SEs have been identified in each cell type; this distribution is widely used to determine the identity of the cells associated with the development of certain diseases. SEs consist of clusters of enhancers densely occupied with master transcription factors and mediator coactivators, as well as other transcription factors and epigenetic regulators at high concentrations (19, 20) (**Figure 1A**). It has become clear that SE deficiency affects the activity of other enhancer regions, resulting in a decrease in SE activity (21). When analyzing the three-dimensional structure of the genome, multiple enhancer regions within one SE have been found to physically interact (22–25). By introducing the concept of SEs, the coordination between transcription factors and multiple enhancer regions was highlighted.

SEs were defined by reinterpreting the results of ChIP-seq experiments. Several SE prediction tools are currently available. However, when looking at the results of previous studies, some of them were not well-documented or required many ChIP-seq datasets. Hence, the best option seems to be the Ranking of Super-Enhancer (ROSE) (19, 26). Here, to identify SEs, all enhancers in a cell type were ranked according to the total background-subtracted ChIP-seq signals of histone modifications, master regulators, and Mediator 1 (Med1), and the total background-subtracted ChIP-seq occupancy was plotted (**Figure 1A**). These plots reveal a clear point in the distribution of enhancers, where the occupancy signal began to increase rapidly. If enhancers have occupancy signals above this threshold, they are supposed to be super-enhancers; enhancers with occupancy signals below that point are considered typical enhancers. Enriched regions of histone H3 lysine (K)-27 acetylation (H3K27ac) were also identified using ChIP-seq data. These domains are ranked according to H3K27ac ChIP-seq signals, and the tangent of the curve was used to define two enhancer populations. However, the enhancer feature H3K27ac alone cannot directly substitute for the master transcription factors and Mediators (20, 27).

### The role of SEs

Interestingly, SEs could provide valuable resources for further studies on cellular control of signals. A catalog of SEs in 86 human cells and tissue types has been generated (20). This analysis identified genes encoding cell-type-specific TFs and candidate master TFs for many cell types that should be useful for understanding cell-type-specific transcription programming. Disease-specific DNA sequence variation is also associated with SE regions (20). Tumor cells acquire SEs at key oncogenes and genes that are hallmarks of cancer development. These studies suggest that SEs guide specific genes essential for many diseases and could be biomarkers for tumor-specific pathologies that could be valuable for diagnosis and therapeutic options. Moreover, the disease-associated expression of microRNAs (miRNAs) is also controlled by SEs (28). Interestingly, miRNA-processors such as Drosha and DGCR8 were also found to occupy SE regions, suggesting that SEs could be platforms for effective RNA processing.

### Enhancer RNAs

In addition, SEs are transcribed to enhancer RNAs (eRNAs), which have been proposed to increase enhancer activity (29). eRNAs are unstable non-coding RNAs whose expression levels correlate with enhancer activity (**Figure 1B**). Because enhancers are tissue- and disease state-specific, eRNAs derived from the same enhancers may differ across tissues (30). eRNAs play an active role in the transcription of nearby genes, potentially facilitating enhancer-promoter interactions *via* loop formation; abnormal eRNA expression is associated with human disease (31–34). Each eRNA may have an independent functional role and some eRNAs may act *in trans*, far from enhancers (35, 36).

## Formation of liquid-liquid phase separation in biological systems

**Figure 2:**
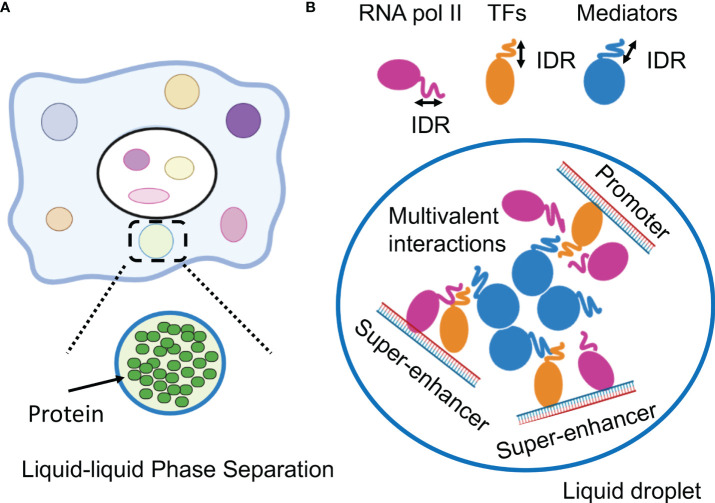
Phase-separation by transcription factors and epigenetic factors. **(A)** Schematic representation of the molecular system that can form the liquid-liquid phase-separation (LLPS). **(B)** LLPS formation by multimolecular complex of transcriptional regulators at super-enhancers for activating gene expression. IDR, intrinsically disordered region.

LLPS has been attracting attention in medical research (6). LLPS is a natural phenomenon observed as oil droplets in water and de-mixing of two liquids when mixing water and oil or shaking a salad dressing. Specifically, LLPS is a physical phenomenon wherein droplets are formed by separating the phases due to the difference in charges between the interacting molecules (**Figure 2A**). Cells have organelles made from membranes consisting of phospholipids. Meanwhile, some compartments are concentrated without a membrane, in the form of granules (37). This enrichment was caused by LLPS, the center of which is RNA-binding protein (RBP) (38). In addition, RNA serves as a seed for defining the locations of phase-separated compartments (39, 40). RBPs function in multiple steps of mRNA splicing from pre-mRNA, such as polyadenylation, nuclear transport, stability maintenance, and localization (39). While RBPs exist in the nucleus as a complex with immature precursor mRNA (pre-mRNA), it also controls the epigenome by binding to long non-coding RNAs (41, 42).

### The role of intrinsically disordered region for LLPS

Importantly, a disordered region of RBPs, IDR, or the low-complexity (LC) domain, facilitates the assembly of LLPS *via* multivalent interactions among nucleotides or amino acids (8, 43). Because IDRs lack a stable tertiary structure and access a wider conformational space, the formation of three-dimensional networks of protein molecules is possible. These regions have low amino acid sequence complexity and enriched in disorder-promoting amino acid residues (Arg, Pro, Gln, Gly, Glu, Ser, Ala, and Lys) without a fixed structure and aromatic residues, such as Phe, Trp and Tyr (44).

The interactions such as cation–pi, pi–pi, electrostatic, and transient cross–pi-contacts are important for the formation of LLPS (8). Recent evidence suggests that the aromatic residues in IDRs are particularly critical for increasing the effect by polarizable pi electron. For example, the cation-pi interactions between Phe and Arg motifs have been proven to be significant for promoting LLPS (45). The Tyr residues in other RBPs such as fused in sarcoma (FUS) can also promote the process of phase separation *in vitro* and in cells (45, 46). IDRs can promote LLPS through weak interactions such as pi-pi interactions mediated by aromatic residues in TAR DNA binding protein 43 (TDP-43) or reversible amyloid-like interactions between TDP-43 and FUS proteins (47). The electrostatic interactions between opposing charge residues of IDRs contribute to the promotion of LLPS. In DNA damage response (DDR) sites, negatively charged poly (ADP-ribose) (PAR) can rapidly recruit positively charged proteins containing IDRs and cause LLPS upon DNA damage (48). In addition, the short stretches of amino acids in IDRs can form localized structures and promote self-interaction. Fibrils formed by short segments of prion-like domains (PrLDs) of RBPs that undergo LLPS have kinked cross–β-sheets termed low-complexity aromatic–rich kinked segments (LARKS) (49). The IDRs located in the C-terminus of the TDP-43 protein contain a structural domain that forms a local α-helix, which enables TDP-43 self-connections and drives LLPS (50). Thus, aggregates are formed by a force that causes a weak bond. It is also known that the formation of granules *via* LLPS is linked to protein aggregation in neurodegenerative diseases such as amyotrophic lateral sclerosis (ALS) (47). Furthermore, in recent years, it has become clear that it works as a physical force that causes various intracellular phenomena, such as autophagy, cell adhesion, and ubiquitination (51).

### Physiological properties of LLPS

Several factors have been shown to modulate physiochemical features of condensates such as the viscoelasticity and condensate structure (7, 8, 52).

### Thermodynamics

The thermodynamics is important physical parameters including a concentration or temperature change can induce phase transitions. For example, stress granule (SG)-formation clustered with FUS and TDP-43 is induced by physiological stress conditions such as heat and oxidative stress (37). As LLPS depens on the concentration of macromolecules, the threshold concentrations are essential factors of LLPS (38). When the concentration of macromolecules in the solution is increased to the limit of solubility, the threshold concentration, the interactions between the macromolecules will become strong and this solution will have propensity to LLPS (52). The threshold concentration is determined by several biophysical parameters including the salt concentration, temperature, and other ions (8). The addition of crowding agent, PEG3000 or glycerol, can also effectively promotes LLPS.

Simple changes to protein sequence can also alter self-assembly of proteins. Lysine and arginine, two positively charged amino acids that can have strikingly different LLPS properties. Arginine can form cation-pi interactions that mediate protein-protein association (53). Poly-Arginine condensates more readily than poly-lysine on a negatively charged nucleic acid scaffold, and is more viscous and aggregation-prone (54).

### RNA

RNAs additionally act as scaffolds for promoting phase transition. RNA regulates the organization of phase separated condensates (8). RNA recruit protein to compete with RNA base paring to promote phase-separation (55). RNAs can not only drive LLPS *via* electrostatic interactions, but repetitive intermolecular base pairing can also produce multivalency and then contribute the formation of LLPS (56).

Considering the role that arginine residues paly in LLPS, the presence of an arginine-rich polypeptide (RRP) induces LLPS of PrLDs of FUS. Whereas RNA can promote LLPS of RRPs but not of PrLDs, suggesting the importance of arginine in forming protein-RNA condensates (57). RNA molecules can differentially partition into condensates as a function of RNA structure, which may help explain why some condensates show spatially patterned RNA localization. RNA sequences affected droplet viscosity, as poly-rA formed more viscous droplets than -rU or -rC (55).

In addition, long non-coding RNAs (lncRNAs) are localized on chromatin and form a cluster of RNA in a particular nuclear region for transcriptional regulation (7, 8). Many findings have demonstrated that NEAT1 lncRNAs can functions as a scaffold RNA and selectively interacts with NONO/SFPQ proteins, which dynamically oligomerize proteins for LLPS to facilitate the assembly of paraspeckle (52).

### Posttranslational modifications

Protein concentration, posttranslational modifications (PTMs) of environmental elements provide a major mechanism for the regulation (7, 52). Emerging evidence suggests that PTMs, such as phosphorylation, acetylation, methylation, and SUMOylation, have important roles in regulating LLPS. PTMs affect the physiological characteristics of regulated amino acids in scaffold protein by diminishing or enhancing the multivalent interactions (8). Thus, PTMs induce a wide range of effects on the structural properties of IDR proteins and drive state changes such as intrinsically disordered states, folded states, dispersed monomeric and phase-separated states (58). For example, Arg methylation mediated by protein arginine methyltransferase (PRMT1) increases the rate of SG formation caused by oxidative stress (59). SUMOylation of PMLs contributes significantly to the formation of the PML nuclear body, whereas de-SUMOylation can lead to a constituent protein being released and nuclear bodies being separated during mitosis (52). Moreover, three distinct IDRs in G3BP1 regulates its properties for enhancing LLPS. This process is fine-tuned by phosphorylation (S149) within the IDRs (60). Thus, PTMs could be a conformational switch that fine-tuned RNA binding and the subsequent LLPS in cells.

### Other factors

Molecular chaperons are critical for controlling the quality and protein homeostasis by avoiding aberrant folding and aggregation. A wide variety of molecular chaperones has been proven to undergo LLPS such as heat shock protein 40 (HSP40) (61). Thermodynamic nonequilibrium processes, such as the hydrolysis of adenosine triphosphate (ATP) can provide free energy to fuel phase separation by facilitating droplet states during LLPS (52). Whereas, ATP is a universal and specific biphasic modulator of LLPS in IDRs, altering physicochemical properties, conformation dynamics, assembly, and aggregation. A high concentration of ATP inhibits the tendency of IDRs in condensate components, solubilizing abnormal, pathological aggregates often associated with neurodegenerative disorders (37).

## Phase separation of transcription factors on SEs

This section outlines the results of recent research on the functional analysis of SEs, focusing on LLPS in gene expression. Recent studies have indicated a direct relationship between gene regulation and LLPS (5, 62, 63). Single-molecule light microscopy determined the spatiotemporal organization of RNA Pol II in the nucleus, revealing that approximately 10–100 RNA pol II molecules form short-lived clusters in the nucleus for several seconds, indicating the presence of transcription factories in living cells (64). This observation provides important evidence for the existence of pre-assembled stable transcription structures. Super-resolution imaging of RNA pol II with a mediator in mouse ES cells demonstrated that hundreds of RNA pol II and mediator molecules formed stable clusters of approximately 10-20 per cell for more than one minute and transient small clusters for approximately 10 seconds (65). Mediators and Pol II are colocalized in stable clusters, which associate with chromatin and have properties as phase-separated condensates in a transcription-dependent manner. In addition, fluorescently tagged mediators and BRD4 (bromodomain-containing 4), a transcriptional and epigenetic regulator, have been found to be locally accumulated in the vicinity of some SEs and have IDRs, which induce LLPS (66). The RNA pol II and FET (FUS/EWS/TAF15) family proteins containing IDRs also undergo LLPS *in vitr* (67, 68). These live-cell imaging studies indicate that transcriptional factories are dynamic structures formed on genes during transcription. Based on these considerations, the hypothesis that TFs, multiple interactions between enhancer regions, and LLPS may be involved in the function of SEs has been investigated (5, 69).

These studies provided a model in which TF clusters formed around the SE activate transcription by transiently interacting with promoters of nearby genes. In these condensates, the important characteristics of aggregates associated with LLPS include: 1) early recovery after the disappearance of clusters due to photobleaching, 2) cluster-to-cluster fusion, and 3) the presence of hydrophobic interactions (66–68). It was clarified that LLPS by transcription factors is strongly induced in the SE region and that complexes comprising TFs, conjugate factors, and mediators connecting enhancers and transcription start sites are formed. The relationship between SE and LLPS was demonstrated by the observation that 1,6-hexanediol (1,6-HD) suppressed SE activity. The aliphatic alcohol 1,6-HD disassembles phase-separated ribonucleoprotein granules and membrane-less structures by disrupting their weak hydrophobic interactions (66, 70, 71). It has been reported that IDR is enriched in many TF proteins and that master TFs form phase-separated condensates with the mediator coactivator at SEs (5, 72). In addition, a repeating sequence undergoes phosphorylation on the C-terminal repeat domain (CTD) of RNA pol II, causing the transcription of DNA to mRNA. Although this sequence is a signal to initiate splicing by RNA-binding proteins, LLPS also functions here (72). Therefore, the transcription mechanism itself could occur without a fixed structure, owing to the weak binding afforded by LLPS (**Figure 2B**).

The cooperative interaction between molecules for aggregation is determined by the number and concentration of interacting factors and the nature of the interaction site, since the presence of IDRs provides an avenue for more interactions. It is assumed that a high concentration of factors works for SE formation while a low concentration works for a typical enhancer. Thus, the SE forms a larger molecular complex than a typical enhancer. Moreover, BRD4 inhibitors targeting acetylated histones and activating transcription suppress SEs more strongly than typical enhancers (26). A rapid change in molecular complex formation was observed in SEs, depending on the number of interaction sites. The synergy between the concentration of the interacting factors and the number of interaction sites determines the sensitivity of SEs to BRD4 inhibition (26).

In the conventional model of gene regulation patterns, an enhancer and promoter physically interact with each other in a one-to-one correspondence to activate a gene. By introducing the concept of LLPS, the relationship between the enhancer and the promoter can be one-to-one or one-to-many, respectively (69). It has been observed that one enhancer can induce multiple genes simultaneously (73). In addition, transcription bursts can be explained by LLPS (74). The temporal formation pattern of the molecular complex is similar to that of the transcription burst, depending on the strength of the enhancer (74). In mammals, the gene expression pattern induced by inflammatory stimuli, such as tumor necrosis factor alpha (TNF-α), is similar to that in a bursting model (75, 76). In the future, it will be determined whether these gene regulation patterns are regulated by LLPS.

## The role of LLPS-mediated TF activation in cancer progression

### FET fusion

Alterations in transcriptional condensates in cancer cells have been reported in recent publications (**Table 1**). These reports highlight the pathological significance of LLPS caused by TFs in the development of cancer. Chimeric fusion TFs produced *via* chromosomal translocation affect the dynamics of LLPS formation and transcriptional activity. Interestingly, FET protein-related translocation and IDRs of the FET RNA-binding protein family are fused to the DNA-binding domains of various TFs, yielding chimeric TFs such as EWS-FLI, EWS-ERG, and FUS-ERG in Ewing sarcoma (87). EWS-FLI TFs recruit the BRG1/BRM-associated factor (BAF) chromatin remodeling complex to the tumor-specific enhancers in a manner dependent on the EWS IDRs and form condensates at GGAA repeat-containing microsatellites (82, 83).

**Table 1:** Summary of transcription factors and epigenetic factors activated by LLPS.

Protein	Cell type	Cellular mechanisms	Reference
YAP, TAZ, TEAD	Lung adenocarcinoma model, melanoma	Induced by IFNγ for anti-PD-1 resistance	77
	Breast cancer	Activation of YAP signaling	78, 79
ERα	Breast cancer	Enhancer activation	80
AR	Prostate cancer	Collaboration with FOXA1 and OCT4	81
FET fusion	Ewing sarcoma	Recruit BAF complex	82, 83
NUP98 fusion	Pediatric AML	Chromatin looping	84, 85
β-catenin	murine embryonic stem cells (ESCs)	Complex with Mediators	86

### β-catenin

Enriched recruitment of signaling factors to enhancers may reflect preferred access to open chromatin and could be associated with activated epigenetic status (13). This binding is mediated by structural changes in the DNA caused by other TFs at these enhancers or by cooperative action through protein-protein interactions with master TFs. However, the mechanism by which a single signaling factor interacts with cell-type-specific enhancers has not been fully elucidated. Signaling factors such as Wnt, TGF-β, and JAK/STAT enter and concentrate in mediator condensates at SEs using their IDRs (86). β-catenin interacts with both condensate components and TFs to selectively activate SE-associated genes. Both condensate and TF interactions contribute to β-catenin localization. These findings suggest that context-dependent transcriptional responses can be achieved using phase-separated condensates and IDRs across cells (86).

### ERα

Moreover, another example of transcriptional machinery reported as a biomolecular condensation on active enhancers is the action of estrogen receptor α (ERα) in response to 17β-estradiol (E2) treatment in breast cancer cells. ERα forms a protein complex, referred to as the “MegaTrans” complex, wherein proteins are recruited at the activated enhancers. This complex is characterized by transcription factors that collaborate with ERα, such as GATA3, FOXA1, and epigenetic enzymes (13). Many components of this complex harbor IDRs. Furthermore, ERα also transcribed high levels of eRNAs in the acutely activated enhancers. These proteins and RNAs exhibit the assembly of RNA-dependent ribonucleoprotein (eRNP) condensates by the physical properties of LLPS at acutely induced enhancers but not at enhancers exposed to chronic stimulation (80). This complex formation and enhancer action were sensitive to chemical disruption using 1,6-HD (66, 71). In addition, a rapid fluorescence recovery of ERα was observed after photobleaching. These results indicate that ERα forms functional condensates to activate enhancers. Moreover, LLPS underlies long-distance interactions and the cooperative activation of acutely induced enhancers (80).

### NUP98 fusion

Recent studies have discovered that nucleoporin-98 and -96 precursor (NUP98)-associated TF chimeras, which are recurrently detected in pediatric acute myeloid leukemia (AML), form nuclear condensates and induce aberrant chromatin looping and leukemogenic gene expression programs (84, 85). In these tumors, the phenylalanine- and glycine-rich IDRs of NUP98, which intrinsically form part of the nuclear pore complex, are fused to various TFs or epigenetic regulators, such as HOXA9, KDM5A, NSD1, DDX10, and PSIP1. These chimeric fusion TFs form nuclear foci in an IDR-dependent manner and interact with various transcriptional regulators. Additionally, the IDRs potentiate the activation of target genes, possibly through increased chromatin binding and retention (84, 85).

### YAP/TAZ/TEAD

The Hippo pathway and the downstream effector Yes-associated protein (YAP) control cell growth, cell fate, and tumor progression. Hippo pathway is important for biological activities such as immune regulation, epithelial homeostasis, and tissue regeneration. YAP and the transcriptional coactivator with PDZ-binding motif (TAZ) are frequently upregulated in cancers that form condensates and promote oncogene expression. Condensates of TAZ were observed at discrete nuclear puncta in breast cancer tissues (78, 79). Hydrophobic interactions are induced by coiled-coil domains of YAP/TAZ for the formation of LLPS (77, 78). Moreover, YAP forms a complex with its transcription factor partner, TEA domain (TEAD) family members, associating with promoters or enhancers to regulate epigenetic conditions and its target genes. Another important role of the YAP pathway is acquiring resistance to cancer immunotherapy (77). Immunotherapy using programmed death 1 (PD-1)/programmed death-ligand 1 (PD-L1) inhibitors has shown promising clinical outcomes in treating many cancer types. However, resistance to immunotherapy is often observed in patients with solid tumors. Blockade of PD-1/PD-L1 activates T cells to induce interferon-γ (IFNγ) production for anti-tumor activity. However, IFNγ mediates adaptive resistance through PD-L1-dependent and -independent mechanisms. LLPS and nuclear translocation of YAP by IFNγ drives a transcriptional program independent of the canonical STAT1-IRF1 pathway (77). In activated enhancers, YAP condensates function as a target gene hub by forming a transcriptional complex with the transcription factor TEAD4, histone acetyltransferase EP300, and Med1. YAP inhibition reduces tumor growth by enhancing the immune response and sensitizing tumor cells to anti-PD-1 therapy.

## Activation and emergence of distinctive transcription factor networks for prostate cancer progression

Changes in transcriptional control mechanisms are associated with the progression of cancer and cancer stem cells, leading to treatment-resistance, recurrence and metastasis (88). This section focus on the transcriptional networks involved in the progression of prostate cancer (PC).

### AR in prostate cancer

PC is the most frequent cancer in men worldwide (89). Although androgen deprivation therapy, anti-androgen receptor (AR) therapy, and taxane-based chemotherapy are effective for advanced types of cancers, many patients with PC develop a more lethal form of PC called castration-resistant PC (CRPC) (90). It is well known that increased activation of androgen receptor (AR) signaling is the primary mechanism in the transition to CRPC (90, 91). Cofactors and collaborating TFs are necessary for AR-regulated gene expression. While coregulators directly bind to the activation function (AF) 1 or 2 domains of the AR, collaborating TFs bind to genomic elements near the AR binding sites. Importantly, some TFs, such as FOXA1, function as pioneer factors that facilitate AR recruitment to target regions through chromatin remodeling (13, 14). Therefore, dysregulation of collaborating TFs can dramatically change the pattern of AR-binding sites between treatment-naïve PC and CRPC (12, 92, 93). AR-binding genes unique to CRPC were not AR-regulated in treatment-naïve PC cells (92–97). Moreover, the ligand-independent splice variant of AR, AR-V7, also facilitates AR activation in PC under castration conditions. These variants are produced by spliceosomal dysregulation in CRPC (98, 99). Thus, targeting epigenetic or chromatin-modeling factors for AR activation may be therapeutically effective against CRPC.

### The role of TFs in lineage plasticity for NEPC development

Notably, long-term AR-targeted treatment causes changes in the properties of cancer cells and dedifferentiates them into more aggressive PC with neuroendocrine characteristics (NEPC) (100) that express neural markers (synaptophedin, chromogranin A, NSE, etc.). NEPC is characterized by low AR expression and can develop from AR-positive tumors. Although there is a theory that AR-negative cells originally existed and proliferated *via* selection (101), it is assumed that the cells themselves change from being AR-dependent to undifferentiated, AR-independent cells. This process, called lineage plasticity, is induced by the increased expression of other TFs, such as N-MYC (102), SOX2 (15), ONECUT2 (OC2) (103, 104), BRN2 (105), and polycomb repressive complex 2 (PRC2) complex EZH2 (102). Other studies have reported that p53 and Rb1 mutations were found in 39% of CRPC of the adenoma subtype. In contrast, these mutations play a role in the progression to NEPC in 74% of NEPC cases. Therefore, it seems that the loss of function of p53 and Rb1 triggers this lineage plasticity (106). Furthermore, attention is being paid to changes of transcription signals due to the increased expression of TFs accompanying the progression of NEPC. Collectively, the high lethality of aggressive PC indicates an urgent need to identify the molecular mechanisms of these TF networks.

## Enhanced phase separation through the collaboration of TFs in prostate cancer

### Collaboration of OCT4 with AR/FOXA1 in prostate cancer

**Figure 3:**
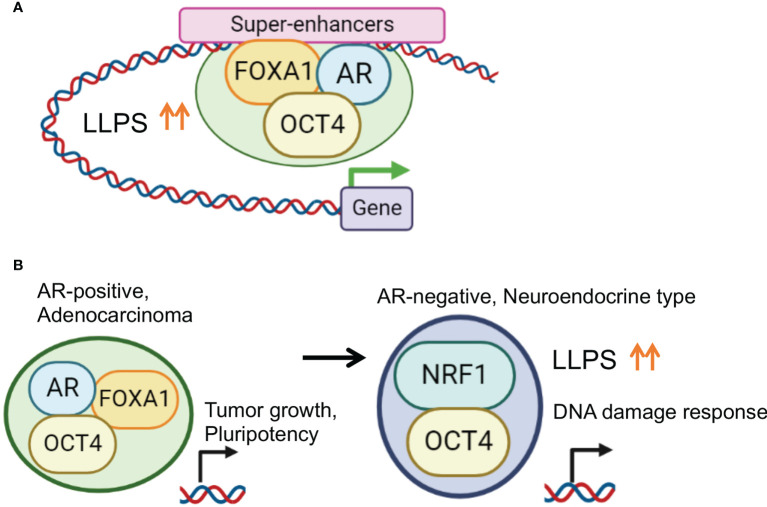
Enhanced phase-separation by transcription factor collaborations for prostate cancer progression. **(A)** OCT4 promotes TF complex formation by enhancing LLPS in PC specific SE regions. In AR positive PC cells, OCT4 interacts with AR, FOXA1 and activates SEs to induce important genes for PC progression. **(B)** Disease specific TF collaboration for PC progression. In AR positive PC cells, OCT4/FOXA1/AR complex regulates genes associated with tumor growth and pluripotency. Repression of AR is frequently observed in NEPC characterized by an aggressive clinical course. In AR negative CRPC cells, OCT4 forms a complex with NRF1 to induce specific target genes associated with DNA damage response for chemotherapy-resistance.

We explored TFs whose expression is upregulated in CRPC compared with hormone-therapy-sensitive PC using the RNA-sequence method (107) and identified OCT4. OCT4 has been reported to be a TF with increased expression in drug-resistant PC tumors (108). OCT4 has two intrinsically disordered activation domains (ADs) responsible for gene activation (5, 109). Stretches of amino acids in the ADs form IDRs which contribute to phase separation. In AR-positive CRPC, OCT4 binding sites overlapped with the binding peaks of AR and FOXA1. Condensates of OCT4 were observed in the nuclei of the PC cells, also suggesting LLPS. Interestingly, motif analysis of OCT4 binding peaks suggests indirect binding of OCT4 through FOXA1 (13, 14) rather than OCT motif-mediated binding. We found that the association of these three factors was necessary for complex formation and transcriptional activation and that the CRPC SE was concentrated at enhancer sites (81). Furthermore, the target genes of AR and OCT4 are enriched with genes related to undifferentiated ability and may be involved in acquiring undifferentiated ability *via* cooperation with AR (**Figure 3**).

Purified and fluorescently tagged AR, FOXA1, and OCT4 proteins were used for an *in vitro* droplet formation assay. Larger condensed droplets were formed by mixing OCT4 and FOXA1/AR proteins compared to AR alone or FOXA1 alone, indicating the OCT-mediated enhancement of the droplet formation ability of the AR complex. Taken together, these results demonstrate that the collaboration of TFs enhances LLPS and droplet formation for transcriptional activation (81).

### The identification of NRF1 as a OCT4 binding partner in AR negative prostate cancer

Of note, another binding partner, nuclear respiratory factor 1 (NRF1), was identified after determining OCT4 binding sites in AR-negative CRPC model cells. NRF1 is a transcription factor involved in the expression of mitochondrial components (110–112) and has an important role in nerves. The interaction between OCT4 and NRF1 was mainly observed in the promoter region. Importantly, enhanced LLPS by complex formation is important for the transcriptional regulation. Interestingly, the target genes regulated by the cooperative action of OCT4 and NRF1 were enriched among genes associated with the DNA damage and repair pathways, suggesting that OCT4 and NRF1 induce drug resistance by modulating the DNA damage response. Thus, the TF network composed of OCT4 is stage-specific, and it is proposed that OCT4 promotes malignant transformation by enhancing LLPS and altering the TF complex (81).

NRF1 as well as OCT4 protein expression is markedly induced in CRPC/NEPC tissues of patients resistant to chemotherapy (81, 108). NRF1 mRNA expression was significantly associated with OCT4 in the NEPC-enriched CRPC cohort and metastatic CRPC cohort, supporting coordinated regulation at the transcriptional level in CRPC/NEPC (81). Taken together, these results indicated that the TF complex of OCT4 is associated with disease progression to lethal PC.

## Targeting LLPS is a promising therapeutic strategy for treatment-resistant cancers

As discussed above, phase separation by TFs at SEs plays a prominent role in diseased cellular states. Therefore, several researchers including us have shown the feasibility of targeting TF-mediated phase separation and subsequent epigenetic regulation as a potent therapeutic strategy for treatment-resistant cancer (52).

### Ribavirin for cancer treatment

In a previous study, *in silico* screening of drugs against PC was performed using Connectivity Map, a database containing the gene expression profiles of different cells treated with various drugs (113). Ribavirin was identified as a candidate drug thought to inhibit the growth of PC tumors with high OCT4 expression. Ribavirin resembles purine RNA nucleotides and prevents the proliferation of RNA viruses, such as the hepatitis C virus, *via* lethal mutagenesis of RNA virus genomes (114). Several studies in cancer cells suggest that ribavirin exerts anti-tumor activity by inhibiting IMP dehydrogenase (IMPDH) and subsequently blocking the *de novo* purine nucleotide synthetic pathway (115, 116) or inhibiting gene expression at the translational level by mimicking the mRNA cap (117). However, the precise molecular mechanism of action of ribavirin in PC remains largely unknown.

### Targeting OCT4-mediated LLPS in prostate cancer

Enhancer/transcriptional activity in OCT4-binding peaks, represented by RNA pol II occupancy, was diminished by ribavirin treatment. Inhibition of AR complex formation with OCT4 and FOXA1 was observed following ribavirin treatment. Moreover, recruitment of the OCT4 and AR complex to AR-binding sites was reduced by ribavirin treatment. These data show that ribavirin inhibits the OCT4-AR axis by modulating OCT4 recruitment. To further analyze the effect of ribavirin treatment on phase separation, we tested whether ribavirin affects droplet formation. The addition of ribavirin reduced the size and number of droplets, suggesting that ribavirin acts on the formed networks of weak protein-protein interactions. Droplet formation is sensitive to high concentrations of salt and ribavirin. More importantly, hormone therapy-resistant PC cells were more sensitive to ribavirin than hormone therapy-naïve PC cells. In xenograft models of AR-positive CRPC, we castrated mice to inhibit androgen action and mimic hormone therapy. Marked inhibition of castration-resistant tumor growth was observed following ribavirin treatment. In a xenograft model of AR-negative PC tumors resistant to cabazitaxel (Cbz), ribavirin sensitized tumors to Cbz treatment. Thus, these results showed growth inhibitory effects to alleviate the aggressiveness of CRPC/NEPC tumors upon combination therapy with ribavirin and chemotherapy (81).

### NSD2 in multiple myeloma

Recent studies have also demonstrated the feasibility of targeting phase separation in cancer treatment (**Table 2**). The development of chemoresistance is the main reason for the failure of clinical management in patients with multiple myeloma (MM). Most patients eventually relapse and often develop multidrug resistance to anti-MM drugs, including proteasome inhibitors (PIs). Therefore, it is critical to elucidate the mechanisms underlying acquired drug resistance in MM (119). Aberrant epigenetic landscapes, such as DNA methylation and histone modifications, contribute to MM progression, clonal heterogeneity, and cellular plasticity (122, 123). Abnormal histone methylation plays an important role in the pathogenesis of MM. High levels of histone methyltransferases and demethylases have been identified in MM patients with genetic mutations (123). Overexpression of a histone methyltransferase called nuclear receptor-binding SET domain protein 2 (NSD2), which mainly mediates histone H3 lysine 36 dimethylation (H3K36me2), activated gene transcription, DNA repair, and cellular survival. NSD2 is an adverse prognostic factor for MM (119).

**Table 2:** Summary of molecules for targeting phase-separation for treating treatment-resistant malignancy.

Protein complex	Molecules/Screning	Molecular function	Tumor model	Ref
HOXB8/FOSL1	GSK-J4 Screening by 303 chemicals	H3K27 demethylase inhibitor	Osteosarcoma	118
SRC3/NSD2	SI-2 High throughput cell-based luciferase assay screening	small molecules interacting with SRC-3	Multiple myeloma	119, 120
OCT4/AR/FOXA1 OCT4/NRF1	Ribavirin *In silico* screening by using Connectivity Map	nuclear analogue	Prostate cancer	81, 113
YAP/TAZ/SRC1	Elvitegravir YAP reporter cell-based screening	integrase inhibitor	Lung cancer	121

### Targeting SRC-3-mediated LLPS for multiple myeloma treatment

Steroid receptor coactivator-3 (SRC-3) is an epigenetic regulator overexpressed in diverse human cancers. The overexpression of SRC-3 is associated with tamoxifen resistance and leads to poor clinical outcomes in breast cancer (120). SRC-3 belongs to the SRC/p160 coactivator family, comprising three members: SRC-1/NCOA1, SRC-2/TIF2/NCOA2, and SRC-3/AIB1/NCOA3. SRC-3 has also been shown to correlate with relapse and poor outcomes in patients with MM. High SRC-3 expression enhances resistance to PI-induced apoptosis. In addition, NSD2 coordinates the elevated SRC-3 expression by enhancing its phase separation and forming a complex with SRC-3 (119). This complex of epigenetic regulators modifies H3K36me2 levels in the promoters of anti-apoptotic genes. Furthermore, the small molecule SI-2, which functions as an SRC-3 inhibitor, blocks the interaction of SRC-3 with NSD2 and represses its activity by disrupting phase separation. Targeting SRC-3 using SI-2 (124) sensitizes PI-resistant MM cells to PI treatment and overcomes drug resistance. Thus, disrupting phase separation by orchestrating the interaction of various epigenetic regulators may be efficacious in overcoming drug resistance.

### Targeting LLPS induced by HOXB8/FOSL1/AP-1 complex in osteosarcoma

In chemotherapy-resistant osteosarcoma, homeobox B8 (HOXB8) and FOS Like 1, AP-1 transcription factor subunit (FOSL1) produce dense and dynamic phase-separated droplets *in vitro* and condensate in the cell nuclei, suggesting phase-separated complex formation (118). Pharmacological inhibition of phase separation in this disease was observed using GSK-J4, an H3K27 demethylase inhibitor. Treatment with GSK-J4 was found to suppress metastasis and recover sensitivity to chemotherapy in osteosarcoma tumors. Thus, these reports provide a phase-separation-based pharmacological strategy by targeting the TF complex, which is a promising treatment regimen for metastatic and chemoresistant malignancies.

### SRC1 in lung cancer

As mentioned above, aberrant YAP/TAZ-mediated transcriptional condensates may contribute to cancer-related pathophysiology (77–79). In lung cancer, siRNA library which contains siRNAs for 15 reported histone acetylation transferase (HAT) was used to perform a screening assay (121). Then it was found that YAP can further form YAP/TEAD/SRC-1 complexes by interacting with SRC-1 and extensively improve YAP transcriptional activity. Elevated expression of SRC-1 in non-small cell lung cancer (NSCLC) was correlated with malignant features and poor prognosis of patients.

### Targeting LLPS induced by YAP/SRC1 complex in lung cancer

Elvitegravir (EVG), one of integrase inhibitors, used to treat HIV infection can suppress cancer metastasis by directly targeting the m6A methyltransferase METTL3 (125). It was newly identified from a YAP reporter cell-based screening by using a library of FDA-approved drugs that EVG inhibits YAP transcriptional activity. Mechanistically, EVG can effectively inhibits the development of the SRC-1/YAP/TEAD complex formation to restrict tumor growth in a YAP-dependent manner by specifically targeting LLPS of SRC-1. Gene expression profiling revealed that EVG suppressed the expression of YAP target genes in lung cancer (121).

### Targeting LLPS pharmacologically in cancer treatment

1,6-HD significantly inhibited cell growth and induced cell death in multiple pancreatic cancer cells (126). The application of 1,6-HD to pancreatic cancer cells can significantly abrogate the LLPS process. 1,6-HD significantly downregulated the expression of a set of genes that were enriched in cytokine-cytokine receptor interactions, WNT signaling pathway, ECM-receptor interaction, MAPK signaling pathway and focal adhesion. Importantly, 1,6-HD downregulate the expression of the MYC oncogene (126). Moreover, researchers have also demonstrated that the anticancer drug melatonin is capable of inhibiting the N-terminal IDR of prion-mediated phase separation in cancer, which results in the alleviation of multidrug resistance (127).

## Future perspective

We demonstrated the function and regulatory mechanism of OCT4-induced TF complex formation through enhanced LLPS on SEs in PC, suggesting that LLPS can be a promising therapeutic target for treatment-resistant cancers. Mounting evidence indicates that LLPS caused by TFs and epigenetic regulators plays a critical role in gene regulation by activating SEs during tumorigenesis. However, there are still other concerns regarding LLPS induced by TF complexes. Although mediator and TF condensates may have occurred before and at the start of gene transcription, it is unclear whether this event depends solely on the characteristics of the IDRs or the presence of multiple enhancer regions in the SE. Moreover, it is still unknown how protein-protein interactions are dependent on RNA or DNA molecules for LLPS formation. In cancer, the activation of SEs due to genomic abnormalities (89) can activate oncogenes, but how phase separation is involved in this event would be clarified in future studies (85, 128, 129). Notably, there is the possibility of therapeutic intervention targeting phase separation to suppress cancer progression. Recent studies (**Table 2**), including ours, have identified several molecules that inhibit phase separation and protein complexes for cancer treatment. However, the precise molecular mechanism underlying this disruption remains unclear. Moreover, the effect of these molecules on the electric charge of IDRs and protein complexes should be investigated.

In conclusion, we propose that repressing TF collaboration involved in the LLPS of SEs could be a promising therapeutic strategy for advanced cancers since it reprograms tumor cells to attenuate cancer progression. Overall, we consider that the model of how transcription occurs and RNA is produced in the genome has entered a new era of change.

## Author contributions

KT conceived the concepts and wrote the first draft of the manuscript. SI revised the first draft. All authors reviewed and approved the final manuscript.

## Funding

This work was supported by grants from the Japan Society for the Promotion of Science (JSPS) KAKENHI (grant numbers 21H04829 (SI) and 20K07350 (KT)); by grants from Takeda Science Foundation (SI and KT), Japan.

## Conflict of interest

The authors declare that the research was conducted in the absence of any commercial or financial relationships that could be construed as a potential conflict of interest.

## Publisher’s note

All claims expressed in this article are solely those of the authors and do not necessarily represent those of their affiliated organizations, or those of the publisher, the editors and the reviewers. Any product that may be evaluated in this article, or claim that may be made by its manufacturer, is not guaranteed or endorsed by the publisher.
